# Lexicon of Dementia: A cross‐linguistic study of the impact of dementia terminology on cultural perceptions

**DOI:** 10.1002/alz.087755

**Published:** 2025-01-09

**Authors:** Mary Chi Michael

**Affiliations:** ^1^ Otsuka America Pharmaceutical Inc., Princeton, NJ USA

## Abstract

**Background:**

Language serves as a powerful tool in shaping perceptions and reinforcing societal norms. Current understanding suggests that the language used to describe dementia can impact how the condition is perceived, understood, and addressed within various cultural contexts. Further, language can play a pivot role in shaping caregiving practices, willingness to seek medical care, and the openness, or lack thereof, to speak about the condition. Understanding these linguistic nuances, both across and within, languages is critical for fostering more effective, inclusive, and tailored approaches to addressing dementia.

**Method:**

To understand how language influences cultural perceptions of dementia, we conducted interviews with linguists, medical anthropologists, health care professionals, care partners, and individuals living with dementia across five languages: Japanese, Mandarin, Swahili, Spanish, and Portuguese. We discussed terminology, regional nuances in language, stigma, misconceptions, care practices, communication strategies, and paths forward for addressing language and improving cultural perceptions. Interviews were supplemented with a comprehensive review of existing literature.

**Result:**

From our research we found that dementia terminology varies greatly across languages. In some, ‘dementia’ carries significant stigma, translating to ‘stupid’, ‘crazy’, ‘senile’, or other, similarly discriminatory terms. In other languages, there is no word for dementia at all, so the local language is mixed with English to describe memory or cognitive decline. Responses are similarly varied. Some cultures are making efforts to, or have already, changed discriminatory language around dementia. Others, particularly those without substantial dementia terminology, look to English to set a better standard.

**Conclusion:**

Across languages, experts concede that dementia terminology is an integral factor in perpetuating stigma, misinformation, and silence. But it doesn’t have to be. Language can also be used to drive positive change. By exploring the connection between language and cultural perceptions, and by studying the measures some have taken to address linguistic stigma, we can uncover valuable insights that inform a broader dialogue on effective and inclusive language for dementia.

